# Effects of sensory integration training on balance function and executive function in children with autism spectrum disorder: evidence from Footscan and fNIRS

**DOI:** 10.3389/fpsyg.2023.1269462

**Published:** 2023-10-25

**Authors:** Junchen Deng, Ting Lei, Xiru Du

**Affiliations:** ^1^College of Sport Arts, Guangzhou Sport University, Guangzhou, China; ^2^College of Graduate, Guangzhou Sport University, Guangzhou, China

**Keywords:** sensory integration training, balance function, executive function, Footscan, fNIRS, Go/No-Go

## Abstract

**Introduction:**

This study investigates the efficacy of sensory integration training (SIT) in enhancing balance and executive functions in children with autism spectrum disorder (ASD), with the aim of highlighting its potential for organizing and processing sensory information in the brain.

**Methods:**

Utilizing Footscan for biomechanical evidence and functional near-infrared spectroscopy (fNIRS) for neural activation, we engaged two participant groups: a control group (*n* = 9) and an experimental group (*n* = 9). Assessments involved the Sharpened Romberg Test (SRT) for balance under varied visual conditions and the Go/No-Go task for executive function.

**Results:**

The SIT intervention significantly improved balance function, particularly in Visual Deprivation (VD) scenarios. Neurophysiological data revealed heightened activation in the right Inferior Frontal Gyrus (R-IFG) and right Middle Frontal Gyrus (R-MFG), suggesting enhanced executive function. The potential of R-IFG/MFG activation as a reliable biomarker for assessing executive function in ASD was identified.

**Discussion:**

The study provides empirical evidence supporting SIT’s effectiveness in enhancing balance and executive functions in children with ASD. The therapy not only improves sensory processing and motor skills but also appears to compensate for sensory deficits, particularly in vision, vestibular perception, and proprioception. Enhanced neural activation in specific PFC regions underscores SIT’s role in improving cognitive aspects, including inhibitory control and cognitive flexibility. The multidisciplinary approach adopted for this research highlights the intricate interplay between sensory-motor functions and cognitive control in ASD, paving the way for integrated therapeutic strategies. Despite these advancements, the mechanisms through which SIT exerts these multifaceted effects require further exploration.

## 1. Introduction

Autism spectrum disorder (ASD) represents the most prevalent neurodevelopmental disorder among children. As per Worldometer’s population statistics, over 70 million individuals globally are diagnosed with ASD, which constitutes 1% of the world’s population, with an exponentially rising incidence rate each year (Worldometer, 2023). Despite ASD’s high prevalence positioning it as a pivotal sociomedical concern (Widiger and Clark, 2000; Hodges et al., 2020), substantial challenges and debates persist surrounding its diagnostic and therapeutic strategies.

From the standpoint of etiology, ASD is broadly considered to possess a pronounced genetic component (Chiocchetti, 2016). The symptomatology of ASD encompasses a range of intricate features, with executive function (EF) impairment (Akbar et al., 2013) and balance function (BF) impairment (Geurts et al., 2009; Golshan et al., 2019) recognized as two primary characteristics in ASD-afflicted children. These manifestations underscore the intricate and multifarious nature of ASD.

Executive function (EF) in ASD is extensively referenced as the foundation of its core challenges (Ozonoff et al., 1991; Russell, 1997). Specifically, EF has implications not just for learning and task execution (Hill, 2004a), but it also further impairs abilities related to social adaptation and independent living (Merchán-Naranjo et al., 2016; Johnston et al., 2019). While EF is documented as a principal symptom of ASD (Lopez et al., 2005), evidence suggests the link between EF and ASD’s core symptoms might not be as straightforward. A study by Jones et al. (2018) discovered that performances in EF tasks don’t directly correlate with measures of social communication or repetitive behaviors, but are indirectly associated via the performance in theory of mind tasks. These insights hint that the neurobiological underpinnings of ASD might be far more intricate than previously perceived. In the realm of neurophysiology, EF deficits primarily manifest in the prefrontal cortex (PFC) (Castelli et al., 2002)—a pivotal brain region integral to decision-making and emotion regulation, Research indicates that such deficits are not merely common in individuals with ASD, but are also intimately linked to their capabilities for social adaptation (Gilbert et al., 2009). Particularly in tasks centered on social and emotional regulation, distinct disparities in PFC neural activity emerge between individuals with ASD and the general populace (Luna et al., 2002). The neurophysiological variations observed might elucidate the challenges noted in social interactions and adaptive behaviors among ASD individuals.

Our study used Go/No-Go tasks to measure executive function in ASD children, especially inhibitory control, which is one of its core components. Executive function refers to a set of higher-order cognitive processes that enable goal-directed behavior, such as planning, problem-solving, working memory, cognitive flexibility, and self-regulation (Daly et al., 2014). Inhibitory control is essential for suppressing irrelevant or inappropriate impulses, responses, or stimuli that interfere with goal attainment (Xiao et al., 2012). Previous studies have shown that ASD children often exhibit impairments in executive function, especially inhibitory control, which may affect their social communication, adaptive behavior, academic performance, and quality of life (Hill, 2004b; Kenworthy et al., 2008; Zaidman-Zait et al., 2017).

Functional near-infrared spectroscopy was employed in this study to monitor neural activation in the prefrontal cortex (PFC) during Go/No-Go tasks. The PFC is a key brain region involved in executive function, as it receives inputs from various sensory modalities and integrates them with internal goals, memories, and emotions (Miller and Cohen, 2001). The PFC also modulates outputs to other brain regions that mediate motor, emotional, and cognitive responses (Fuster, 2008). Previous studies have used fMRI or EEG to investigate neural correlates of executive function in ASD children and found abnormal activation patterns in the PFC and other brain regions, such as the anterior cingulate cortex (ACC), the inferior parietal lobule (IPL), and the cerebellum (Schmitz et al., 2006; Solomon et al., 2009; Christakou et al., 2013).

The significance of balance function is frequently underemphasized in ASD treatment and research. Notably, when juxtaposed with typical development (TD), those with ASD clearly demonstrate unique challenges in balance function, encompassing impediments across sensory processing, motor coordination, and executive function (Fournier et al., 2010a). More crucially, balance function deficits not only curtail ASD patients’ prowess in fundamental motor skills and self-care capabilities, but might also have profound adverse ramifications for their social engagement and holistic cognitive growth (Smith et al., 2012; Bal et al., 2015). Additionally, recent research further unveils the intricate nature of balance function within ASD. Notably, when considering the relationship between balance function and the function of vestibular organs, their interrelation is even more pronounced. In terms of vestibular functional disturbances in ASD, the primary symptoms are postural instability, dysfunctional gait, and hindered gaze. To sustain postural stability and typical balance, real-time and precise acquisition and processing of visual, somatosensory, and vestibular inputs are imperative, underscoring the central role of vestibular organs within ASD (Mansour et al., 2021).

Extensive research has demonstrated that children with autism experience sensory integration disorders, which impact their daily life skills (Doumas et al., 2016). Dr. A. Jean Ayres’ sensory integration training (SIT) remains a predominant measure in ASD movement interventions primarily aiding ASD individuals in enhancing the processing and integration of sensory information (Lang et al., 2012). Through the improvement of balance, executive functions, and posture control in ASD patients, SIT has been shown to elevate sensory discrimination and neural assimilation. While the foundational evidence for SIT exhibits inconsistencies, a growing body of scholars delves into its efficacy. Yet, most investigations remain at the systematic review level, lacking interdisciplinary cross-verification, particularly concerning neuroscience evidence (Kilroy et al., 2019; Schoen et al., 2019).

Within the holistic approach to ASD treatment, SIT stands as a central intervention strategy. The growing demand for nuanced evaluations and tailored treatment strategies underscores the essential role of pioneering diagnostic tools and methodologies. Set against this backdrop, the integration of Footscan for plantar pressure and balance analysis, along with fNIRS for frontal lobe functional imaging, marks a pivotal advancement. Footscan offers precise measurements of plantar pressure and delves deeper into balance and posture control (Balasubramaniam and Wing, 2002). These elements are integral to addressing the prevalent balance deficiencies observed in ASD patients. Leveraging biomechanics, Footscan adeptly pinpoints and quantifies nuanced variations in balance and posture control (Fournier et al., 2010b), delivering essential insights for tailoring SIT interventions. Concurrently, as an avant-garde neurophysiological instrument, fNIRS facilitates real-time monitoring of PFC neural dynamics, shedding profound light on the EF challenges inherent in ASD patients (Singh et al., 2005). These tools not only enrich our comprehension of ASD’s intricate manifestations but also amplify the customization and efficacy of therapeutic approaches, underscoring biomechanics and neurophysiology’s pivotal roles in holistic ASD treatment. This groundbreaking methodology accentuates the importance and necessity of innovative solutions to multifaceted challenges in ASD therapy, forging a robust foundation for future profound research and implementations.

## 2. Materials and methods

### 2.1. Participants

**FIGURE 1:**
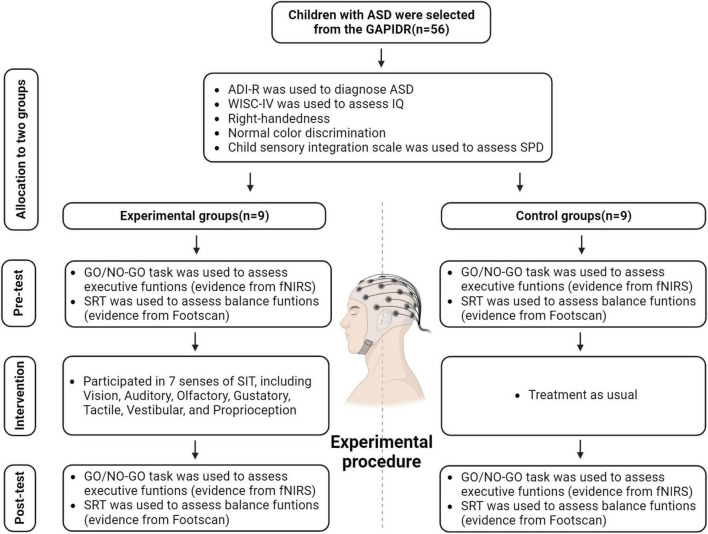
Enrollment, pre-test, intervention, and post-test procedures.

The study was conducted in accordance with the Declaration of Helsinki, and approved by the Human Experimental Ethics Inspection of Guangzhou Sport University (ethics number: 2022LCLL-21, date of approval: 7 July 2022). At the outset, the study recruited 56 ASD patients aged between 6 and 11 years, all hailing from the Guangzhou Association of Persons with Intellectual Disabilities and Their Relatives (GAPIDR). Owing to the inherent heterogeneity of ASD (Lai et al., 2014), and to ensure experimental data remained within pertinent observational parameters, participants underwent preliminary screenings based on their IQ, the severity of their autism, among other factors (Figure 1).

We screened children for inclusion in the study based on the following criteria:

(1) Diagnosis of ASD (aged 4–11 years) by a tertiary hospital. Participant ASD diagnoses were substantiated using the Chinese edition of the Autism Diagnostic Interview-Revised (ADI-R) (Rutter et al., 2013). Given our study’s emphasis on a range of non-social cognition assessments, we excluded participants with low-functioning ASD. We assessed all participants using the fourth edition of the Chinese Wechsler Intelligence Scale for Children (WISC-IV) (Wechsler, 2003; Zhang, 2009) excluding those with an IQ score below 80. Prior studies suggest that intelligence might modulate cognitive performance in ASD; moreover, cognitive evaluations and training appear to be less effective for ASD children possessing below-average intelligence (Rommelse et al., 2015). For these particular interview segments, we engaged a clinical psychologist—unfamiliar with the study’s content and hypothesis—from the GZSU Sports Medicine Rehabilitation Center.
(2) No prior participation in similar research.
(3) Right-handedness. Hauck and Dewey (2001) found that autistic children with a distinct hand preference outperformed their counterparts without such a preference in motor, linguistic, and cognitive tasks (Hauck and Dewey, 2001). To mitigate any disparity affecting our experiment’s outcomes, we specifically chose right-handed participants, instructing them to consistently use their right-hand middle finger for the Go/No-Go task.
(4) Normal color discrimination. In assessing participants’ executive functions, our study utilized two psychological research paradigms: the Stroop color-word task and the Go/No-Go task. But in the pilot phase, participants exhibited an error rate surpassing 30% in the Stroop test, which meant our data didn’t align with anticipated outcomes. As a result, we opted for the Go/No-Go task as described by Monden et al. (2015) to serve as our study’s psychological research framework (Monden et al., 2015). During participant selection, though, we ensured they met the criterion of having normal color discrimination abilities.
(5) No alcohol consumption, late sleeping, or engagement in high-intensity sports immediately prior to the experiment.
(6) Exclusion of data with error rates higher than 30% for Go/No-Go tasks during the analysis.
(7) Exclusion of data with head motion noise in cerebral blood oxygen signal data from fNIRS exceeding the critical value.

Initially, we identified 20 potential participants whose mother tongue was Chinese. Guardians of these participants were asked to read and sign informed consent forms, detailing specifics of the experiment, prior to participation.

### 2.2. Children sensory processing disorders scale

Over the last thirty years, Ayres developed a collection of clinical assessments focused on sensory integration. For each subtype of sensory integration dysfunction, checklists were established (Ayres, 2005), to be completed by parents. These evaluations were then used to assess the severity of sensory integration issues in children. Reflecting the cultural context of China, Jung (2018) of Taiwan amalgamated various symptom checklists to create a consolidated sensory integration checklist (Jung, 2018). Researchers from the Mental Health Institute of Beijing Medical University, such as Ren et al. (1994), imported the Child Sensory Integration Scale (Ren et al., 1994) from Taiwan’s Child Brain Development Alliance. Initially, they conducted trials in the Beijing region, and subsequently expanded their testing to 14 provinces and cities nationwide, establishing a standard model. For this scale, age-adjusted standard scores were chosen as the scoring metric, ensuring both accuracy and objectivity in the assessment results. The scale’s scoring is straightforward, allowing examiners to understand and use it effortlessly. Moreover, since the scale’s questions relate to scenarios seen in children’s everyday life, parents find them easy to answer. Such features highlight the scale’s strong applicability and acceptability. The sensory integration scale is validated for its objectivity and utility. It’s effective in evaluating the developmental progress of children’s sensory integration capabilities, gauging the severity of sensory integration disorders, and serving as a benchmark to compare the efficacy of sensory integration treatments. With a test-retest reliability between 0.48 and 0.74, and structural validity from 0.48 to 0.93, the scale exhibits robust reliability and validity. This attests to its strong usability and acceptability in mainland China. In our research, the scale’s Cronbach’s α is 0.802.

The “Child Sensory Integration Scale” is designed to evaluate sensory integration development in school-aged children aged 2–11 years. This scale encompasses 5 factors and 58 items in total, addressing vestibular sensation, tactile perception, proprioception, learning abilities, and issues specific to children aged 10 and older. Given that our study primarily involves children under 10, the last factor was excluded. The scores from each item on the scale, when combined with the age of the participants, are converted into standard scores. A score below 40 indicates a mild sensory integration disorder, while a score below 30 suggests a severe disorder. The scale utilizes a 5-point Likert scoring system, ranging from “never” (highest score) to “always” (lowest score). Parents or knowledgeable individuals should diligently complete the scale based on the child’s behavior in the most recent month. The four items of the scale are as follows:

(1) Gross Motor and Balance: This primarily focuses on the body’s major motor abilities, encompassing 14 items like “clumsiness with hands and feet, and being prone to falls.”
(2) Tactile Over-Defensiveness and Emotional Instability: This section assesses emotional steadiness and heightened defensive behaviors, featuring 21 items such as “shyness, unease, a penchant for solitude, reluctance to socialize, being easily overwhelmed by TV or stories, and tendencies to shout or laugh abruptly.”
(3) Proprioceptive Challenges and Coordination Difficulties: Mainly concerns the body’s proprioceptive and balance coordination abilities, including 12 items such as “slow actions in dressing and tying shoelaces; disliking somersaults, rolling over, and climbing heights.”
(4) Learning Disabilities or Coordination Issues: This section addresses the learning challenges arising from sensory integration difficulties, featuring 8 items such as “frequent word skipping during reading, omissions during copying, misordered writing strokes, distractibility, restlessness, constant in-class distractions, challenges in meeting teachers’ expectations, and recurrent significant setbacks.”

Upon application of a sensory integration rating scale, we found 18 of the 20 eligible ASD children showed symptoms of Sensory Processing Disorders (SPD) (Table 1). This prevalence aligns with Professor Yi Shin Chang’s (University of California, San Francisco) research indicating over 90% of children with ASD demonstrate SPD symptoms (Chang et al., 2014). The remaining 18 participants were randomly divided into equal experimental and control groups (*n* = 9 per group).

**TABLE 1:** Baseline physical indices and parameters of participants prior to the experimental intervention.

Group	N (male/female)	Age (years)	BMI (kg/m^2^)	Skeletal muscle (kg)	SPD
Control	9 (7/2)	6.22 ± 0.97	16.11 ± 2.42	14.42 ± 2.01	21.89 ± 5.23
Experimental	9 (7/2)	6.56 ± 1.42	15.40 ± 2.26	13.41 ± 1.86	22.56 ± 4.48
F		1.888	0.162	0.131	0.552
p		0.570	0.529	0.284	0.775

### 2.3. Footscan measures of Sharpened Romberg Test (SRT)

We utilized the Footscan plantar pressure and balance analyzer, a widely employed Center of Pressure (COP) analyzer and balance analysis system in motor biomechanics. The Footscan system consists of a 50 cm × 40 cm pressure plate system and balance ability analysis software. The pressure plate contains 4 pressure sensors per square centimeter, totaling approximately 4,000 sensors, which can effectively depict pressure distribution under both stationary and dynamic conditions. The software generates a detailed plantar pressure distribution map with multi-dimensional coloring, which offers a comprehensive overview and quantitative measurements of pressure distribution (Yu et al., 2022).

Participants were instructed to stand on the Footscan and perform the Sharpened Romberg Test (SRT) to gather data on their balance capabilities. The Sharpened or Tandem Romberg test is a variation of the original test (Black et al., 1982; Johnson et al., 2005). By analyzing participants’ responses during the SRT and the biomechanical feedback from Footscan, we offered biomechanical insights into the effects of SIT on the balance function in children with ASD. The strength of the SRT lies in its role as an enhanced static balance assessment task. It can heighten the participants’ bodily control and focus during the task (Fournier et al., 2010b), and the increased challenge in posture control can partially showcase the participants’ executive functions (Smith et al., 2021). The Romberg Test is a tool designed to evaluate individuals’ sense of balance, particularly assessing the function of the spinal cord’s dorsal column. It serves as a diagnostic instrument for sensory ataxia (Nguyen et al., 2018), encompassing conditions such as subacute combined degeneration of the spinal cord, posterior cord syndrome, and spinal cord hemisection (Brown-Séquard syndrome). The test has been recognized as a sensitive and precise measure for evaluating imbalances due to central vertigo, peripheral vertigo, and head injuries, and its clinical application spans nearly 150 years (Lanska and Goetz, 2000; Forbes et al., 2023). Nevertheless, our objective in employing the SRT wasn’t to clinically diagnose participants. Instead, we aimed to have them adopt a scientifically sound standing posture for data collection on Footscan, thereby bolstering the scientific rigor of our experimental data.

**FIGURE 2:**
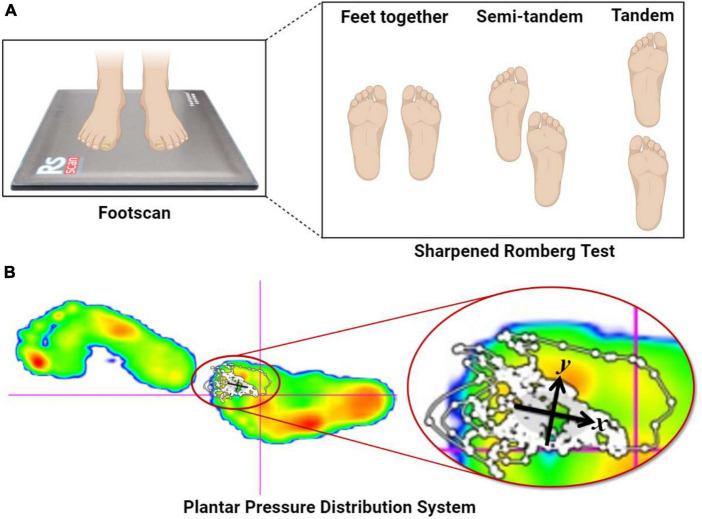
Sharpened Romberg Test and plantar pressure distribution system. **(A)** The SRT balance assessment comprises three stances: feet together, semi-tandem, and tandem. **(B)** The Footscan device from Rscan Company collected COP*_*x*_*, COP*_*y*_*, TTW, and EA when the participants’ feet were stationary and upright. COP*_*x*_* and COP*_*y*_* represent the displacement distances in the *X*- and *Y*-axis directions, respectively, caused by body offset during SRT, and are negatively correlated with balance ability. TTW denotes the total length of the moving trail generated by COP*_*x*_* and COP*_*y*_* during SRT tasks and is also negatively correlated with balance ability. Lastly, EA signifies the elliptical area surrounding 95% of the COP moving trail generated during SRT and is negatively correlated with balance ability (Mikolaizak et al., 2017).

The SRT balance assessment comprises three stances: feet together, semi-tandem, and tandem. In this study, we utilized the tandem stance of the SRT to capture participants’ balance metrics on Footscan. During the SRT balance assessment, participants were instructed to remove their shoes and stand on the Footscan device. They were required to align their feet precisely from heel to toe, cross their arms over the chest, rest their open palms on the respective shoulders, and maintain their chin parallel to the ground. Once the participant achieved a stable posture, the official task commenced. The experiment integrated two conditions: Visual Deprivation (VD) and Normal Visual (NV), with a duration threshold set at 30 s for each test (Figure 2).

All participants underwent three testing phases. In the first phase, guardians provided the participants’ basic information and signed the experiment’s informed consent forms. In the second phase, participants completed a Footscan pre-test. Here, participants were familiarised with the testing procedure, which was formally executed after a practice run. In the final phase, within 2 days of concluding the intervention, participants completed post-test data collection with Footscan. All testing sessions were scheduled between 3:00 and 5:00 PM.

### 2.4. Functional near-infrared spectroscopy (fNIRS) measures of cortical activity

#### 2.4.1. Multimodal Go/No-Go tasks

**FIGURE 3:**
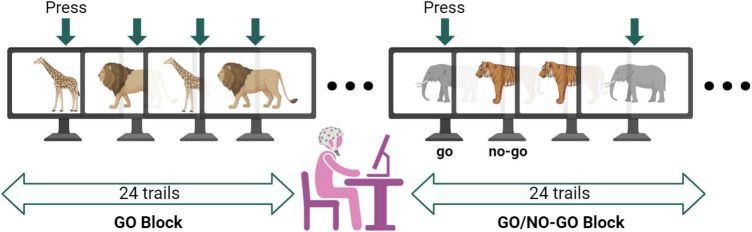
Diagram of Go/No-Go testing conditions. The entire Go/No-Go task consisted of 48 trials, with 24 visual stimulus pictures for both the GO and Go/No-Go tasks. The target stimulus was presented randomly, with all visual stimuli having a duration of 1000 ms. The fixation point was represented as “+.” The interval between each visual stimulus was random, either 400, 600, or 200 ms. Participants were required to make judgments on the presented visual stimuli − pressing the space bar for Go stimuli and not responding to No-Go stimuli, with all keys pressed using the right hand. A practice session was conducted before the formal experiment, and the formal experiment was only initiated when the accuracy ratio (AR) reached 85%. Data with a task AR lower than 85% was discarded.

In this study, we used the classical paradigm of Go/No-Go tasks to assess executive function in our participants (Verbruggen and Logan, 2008; Monden et al., 2015; McKay et al., 2021). These tasks have been validated in previous experiments evaluating functional outcomes in ASD. The Go/No-Go tasks comprise two major blocks, GO and Go/No-Go, with each block containing 24 trials. During the GO tasks, participants were shown pictures of two animals (giraffes and lions) randomly and were instructed to quickly press the space bar upon seeing the animal pictures. During the Go/No-Go tasks, participants were shown pictures of two animals (tigers and elephants) randomly. They were instructed to quickly press the space bar when they saw the elephant, with 50% of the trials evoked by NO-GO stimuli (Figure 3).

All participants had to complete three tests. First, basic information was collected from the participants, and the experiment’s informed consent form was reconfirmed with the guardian. Second, a pre-test of the Go/No-Go tasks was completed. participants were given instructions on the requirements and methods of the Go/No-Go tasks and a practice session before the actual experiment. Third, consistent with the Footscan test scheme, post-test data collection was completed within 2 days after the intervention. During the Go/No-Go tasks, participants wore the fNIRS optode cap, and blood oxygen signal data from the brain’s prefrontal cortex region were collected during the pre- and post-tests.

#### 2.4.2. fNIRS parameter information and image processing

The experiment employed a dual-wavelength fNIRS system (695 and 830 nm; ETG-4000, Hitachi Medical Corporation, Tokyo, Japan) to monitor cerebral hemodynamics changes during the completion of Go/No-Go tasks. fNIRS primarily detects changes in hemoglobin in the participants’ prefrontal cortex using the emission aurora on the optode cap, thereby analyzing the degree of nerve activation in this brain region. The detection panels of the dual-wavelength optode cap were arranged in a 3 × 5 grid. Each panel comprised eight emitting and receiving optical machines, spaced 3 cm apart from each other. The midpoint measurement region between an emitting and receiving optical machine pair was termed a channel. Each panel contained 22 channels (CH).

For the spatial analysis of fNIRS data, we adopted virtual spatial coordinate registration methods to register fNIRS data in Montreal Neurological Institute (MNI) spatial coordinates. This approach allowed us to match the location of the brain region of each measuring channel with the molecular brain imaging (MRI) database and present it in the computer as functional imaging (Singh et al., 2005). During data analysis, the oxyhemoglobin (oxy-Hb) and deoxyhemoglobin (deoxy-Hb) signals of each channel were preprocessed separately using first-order polynomial fitting and a high-pass filter. A 0.01 Hz cutoff frequency was used to correct for previous errors, and a 0.08 Hz low-pass filter was utilized to eliminate heartbeat pulsation, reducing artifacts caused by non-medium substances such as scalp hair and oils on the measuring channel signal. If signal fluctuation occurred repeatedly during the measurement process, experimenters would mark and record this channel and modify or eliminate the channel data in later data analysis.

Finally, during data output and analysis, the exported optical data was solved according to the Beer-Lambert law, which can effectively reflect changes in blood oxygen signals of oxy-Hb, deoxy-Hb, and total hemoglobin (Hb). During data analysis, particular emphasis was placed on the signal changes of oxy-Hb, as its sensitivity, signal-to-noise ratio, and test-retest reliability to changes in cerebral blood flow were all higher compared to deoxy-Hb and total Hb.

### 2.5. The SIT intervention scheme

The SPD screening process dictated the intervention scheme for the participants, based on group classifications. The experimental group received sensory integration therapy (SIT) (Schaaf and Mailloux, 2015) as an intervention for 8 weeks, while the control group was assigned to the usual treatment. During the formal intervention process, all participants wore heart rate monitors to maintain a heart rate within 50–69% HRmax, achieving moderate to low-intensity aerobic exercise. However, these data weren’t analyzed. As a rule, participants were not permitted to partake in regular exercise outside of the experiment, or their data would be discarded. This requirement was discussed and agreed upon with the guardians before the experiment, with detailed explanations provided. We checked each participant’s daily condition and physical feelings via oral inquiries before each intervention session. The sensory integration therapist for the experimental group hailed from the GZSU Sports Medicine Rehabilitation Center. This therapist’s senior certification in sensory integration was co-endorsed by the National Autism Rehabilitation Research Center under the China Rehabilitation Research Center (CRRC) and the Beijing Association for Rehabilitation of Autistic Children. The therapist was not briefed on the specific details or hypotheses of the study and solely performed professional sensory integration therapies. The therapeutic sessions occurred in a sensory integration therapy room, outfitted with equipment essential for SIT. The intervention strategies adhered to the SIT principles set by Ayres (2005), which have since been substantiated by a plethora of scholars (Parham et al., 2007, 2011; Schaaf et al., 2014). The SIT intervention specifically encompasses the subsequent seven sensory systems:

(1) Vision System. A serene and comfortable visual setting is established using tools such as a dark room or blackout curtains, minimizing external distractions. Within the confines of a dark room or behind blackout curtains, tools like colored lights or projectors facilitate incremental visual stimulation. Participants are exposed to variations in colors, shapes, and sizes, allowing them to perceive a spectrum of visual experiences. Through these exercises, participants hone their skills in coordinating eye movements and in spatial visual perception—tasks may include tracking moving entities and distinguishing an object’s position, orientation, and distance. Emphasis during these exercises is on coordinated eye movements and spatial visual perceptions, including tracking moving entities and pinpointing an object’s position, direction, and distance. Care is taken during the sessions to manage the duration and intensity of visual stimuli, ensuring that there’s no overstimulation or resultant eye strain. Concurrently, participants are provided with positive reinforcement and guidance, incorporating methods like encouragement, questioning, and direction. This approach seeks to diminish any heightened sensitivity or aversion they might have toward visual stimuli.
(2) Auditory system. Through the use of tools like headphones or speakers to play sounds of different types and intensities, such as music, noise, speech, etc., for progressive auditory stimulation activities. The exercises involve modulating aspects like the volume, rhythm, and frequency of sounds, enabling participants to discern varied auditory sensations. Within these activities, they exhibit auditory discernment and listening abilities, such as identifying the origin, content, and significance of sounds. During these sessions, meticulous attention is given to the duration and strength of auditory stimuli, ensuring there’s no overstimulation or resultant ear discomfort. At the same time, participants receive positive reinforcement and feedback, like praise, demonstration, and correction, to enhance their auditory system’s discrimination and language listening capabilities.
(3) Proprioceptive System. Leveraging tools such as resistance balls, punching bags, and dumbbells, exercises are conducted to stimulate the proprioceptive sense, emphasizing both force and direction. Participants engage in actions like pushing, pulling, pressing, throwing, and pinching, enabling them to become aware of their muscular strength and the agility of their joints. Within these exercises, participants hone their discernment and perception skills, tasks may encompass gauging force intensity, determining directional orientation (forwards or backwards), and evaluating positional altitude (higher or lower). Throughout the sessions, emphasis is placed on guiding participants to employ movements with appropriate strength and trajectory, ensuring that there’s no undue exertion or risk of harm to either themselves or others. At the same time, participants are offered positive reinforcement and feedback—including methods like commendation, demonstrations, and corrections—with the objective of augmenting their motor control abilities and fostering self-awareness.
(4) Tactile System. Materials such as towels, blankets, and pillows are employed to craft a gentle and cozy tactile ambiance for participants, mitigating undue tactile sensations and stress. Within this soothing tactile setting, tools like massage balls, acupressure boards, and tactile balance platforms facilitate gradual enhancements in tactile stimulation exercises. Engagements like massaging, stimulating, and balancing techniques are incorporated, granting participants exposure to a spectrum of tactile sensations spanning multiple intensities and depths. Within these exercises, participants refine their tactile discernment and awareness, undertaking tasks like differentiating object textures, temperatures, and forms. Emphasis is placed on moderating the duration and potency of tactile stimuli throughout these sessions, ensuring that participants aren’t overwhelmed or left with any skin irritations. At the same time, participants receive positive reinforcement and cues, such as praise and hugs, to increase their adaptability and satisfaction with tactile stimulation.
(5) Olfactory System. Tools like aromatherapy lamps, perfumes, and banana peels are harnessed to craft a fragrant and refreshing olfactory ambiance for participants, elevating olfactory stimuli and evoking delight. Within this aromatic setting, a variety of scented items or edibles are introduced. Progressive olfactory stimulation activities, like savoring fragrances or guessing specific scents, are undertaken. Participants are immersed in an array of odors, each differing in kind and intensity, engaging them in olfactory discernment and memory exercises throughout. This includes tasks like distinguishing the source, identifying the name, or characterizing specific scents. Throughout these sessions, there’s a concerted effort to modulate the duration and potency of olfactory stimuli, ensuring participants are not overwhelmed or left with any nasal discomfort. At the same time, participants are given positive reinforcements and feedback, such as praise, queries, or guidance to enhance their discernment and retention capacities in relation to olfactory stimuli.
(6) Gustatory System. Offer participants a variety of foods or beverages, creating a rich and diverse gustatory environment to enhance taste stimulation and preferences. Within this flavorful and diverse setting, introduce foods or beverages of varying flavors. Engage participants in progressive taste-based activities like sampling, comparing, and selecting. This enables them to discern different tastes and intensities, and to differentiate aspects like the names, ingredients, and textures of the foods or beverages. Throughout the activities, carefully regulate the duration and strength of taste stimuli to avoid overwhelming the senses or causing discomfort in the mouth. Concurrently, provide participants with positive reinforcement and guidance, like commendations, inquiries, and direction, bolstering their capacity to discern and develop preferences for various taste stimuli.
(7) Vestibular System. Utilizing tools like swings, scooters, and jump ropes, craft a dynamic and engaging vestibular environment to amplify vestibular stimulation and interest for participants. Within the lively and engaging vestibular setting, employ tools like swings, scooters, and jump ropes for incremental vestibular stimulation activities. Introduce actions like rocking, spinning, and jumping to let participants experience movements of varied directions, speeds, and magnitudes. This promotes the development and display of balance and spatial orientation skills, such as stance maintenance, posture adjustment, and visual tracking. Throughout the activities, ensure careful regulation of the vestibular stimuli’s duration and strength, so as to prevent overwhelming sensations or inducing fear and discomfort. Concurrently, furnish participants with positive reinforcement and encouragement, including commendations and embraces, to bolster their tolerance and adaptive reactions to vestibular stimuli.

Implementation Feedback. Administer sensory integration therapy three times weekly, with each session spanning 60 min. Allocate 10–15 min to each sensory system. The therapy regimen extends over 8 weeks. Document participants’ responses and advancements for every activity. Adjust activity challenges and intensities as dictated by observed outcomes. Maintain regular communication with parents and teachers, gaining insights into participants’ behavior at home and school. Offer guidance and support based on these insights.

### 2.6. Statistical analysis

Experimental data were analyzed using SPSS Statistics 22. Repeated measure ANOVA was used to test the main effects and interactions of the Go/No-Go cognitive neural tasks. For reaction time (RT), accuracy ratio (AR) of Go/No-Go effects, blood oxygen signal differences, and the SRT, we combined visual display (VD) task conditions to collect data on COPx, COPy, TTW, and EA. A *t*-test was performed pre- and post-experiment to analyze data differences, using repeated measure ANOVA as a basis.

Out of 18 eligible ASD children screened for SPD using the Children’s Sensory Integration Rating Scale, 10 showed moderate SPD symptoms, six had mild SPD symptoms, and two were normal. The 18 SPD participants were randomly divided into control and experimental groups, nine in each. There were no statistical differences between the two groups in terms of age, sex, BMI, and skeletal muscle mass (*P* > 0.05, Table 1), suggesting that all participants met the intervention conditions for this experiment.

## 3. Results

### 3.1. Results from Footscan measures of Sharpened Romberg Test (SRT)

The centre of pressure (COP), the instantaneous locus of forces applied and counteracted during the contact between the human body’s sole and the ground, primarily navigates the heel-to-toe range when the body is standing. The displacement remains relatively minimal (Cornwall and McPoil, 2000). The projection of the COP path, which consists of the entire instantaneous locus, onto the Footscan force-measuring platform, forms a gait line (Fuller, 1999). The Footscan employs a resistive pressure-sensitive sensor in an X-Y matrix, capable of recording real-time pressure data from participants. This data is further processed and visualized via Footscan 9 to extract measurement characteristics.

Data related to the SRT was pre-processed for both control and experimental groups, collected before and after the experiment (Table 2). Following an 8-week intervention, very significant differences appeared in COPx, COPy, TTW, and EA across the NV and VD tasks within the experimental group. Lower TTW and EA values indicate superior static balance ability. Consequently, these findings demonstrate that sensory integration training (SIT) can significantly enhance ASD children’s balance function.

**TABLE 2:** Physical indices and parameters of participants prior to the experimental intervention.

		Control group		Experimental group	
Index		Pre (M ± SD)	Post (M ± SD)	Pre (M ± SD)	Post (M ± SD)
NV SRT	COPx/mm	22.22 ± 3.46	19.11 ± 3.06	23.44 ± 3.13	17.44 ± 2.88
	COPy/mm	21.22 ± 2.77	17.78 ± 1.79	22.56 ± 3.13	16.22 ± 2.05
	TTW/mm	1882.44 ± 255.29	1615.00 ± 207.65	1846.33 ± 308.59	1361.00 ± 102.33
	EA/mm2	1391.44 ± 258.44	1103.67 ± 121.06	1395.33 ± 150.69	920.22 ± 39.15
VD SRT	COPx/mm	25.33 ± 2.60	24.44 ± 2.30	24.56 ± 3.00	18.33 ± 1.73
	COPy/mm	22.11 ± 2.26	21.33 ± 0.87	26.22 ± 2.11	17.89 ± 2.20
	TTW/mm	2574.44 ± 221.29	2341.78 ± 261.39	2717.33 ± 173.80	2427.00 ± 179.66
	EA/mm2	1766.22 ± 139.09	1600.56 ± 111.99	1845.67 ± 145.98	1691.33 ± 108.27

COP_xy_, Distance traversed by the Centre of Pressure (COP) on the x- and y-axis during upright standing; TTW, Total length of the COP’s moving trail; EA, elliptical area encapsulating 95% of the COP’s moving trail; “Pre” refers to pre-test data, and “Post” indicates post-test data.

**FIGURE 4:**
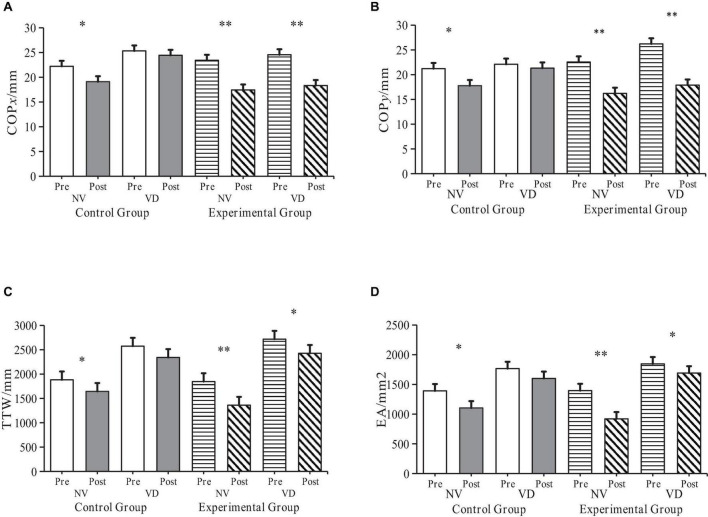
Alterations in ASD children’s balance ability in control and experimental groups pre- and post-intervention. “NV” represents Normal Visual, “VD” represents Visual Deprivation; *p < 0.05, **p < 0.01. COP*_*xy*_*: Distance traversed by the Centre of Pressure (COP) on the *x*- and *y*-axis during upright standing; TTW: Total length of the COP’s moving trail; EA: Elliptical area encapsulating 95% of the COP’s moving trail. **(A)** COPx Changes of participants before and after intervention. **(B)** COPy Changes of participants before and after intervention. **(C)** TTW changes of participants before and after intervention. **(D)** EA changes of participants before and after intervention.

The data gathered for COPx, COPy, TTW, and EA during NV and VD tests before and after the intervention was dynamically compared for both control and experimental groups (Figure 4). The *t*-test results showed that under NV conditions, all four indices exhibited significant differences in the control group (*P* < 0.05), and even more significant ones in the experimental group (*P* < 0.01).

Building upon the descriptive statistics and the *t*-test, a repetitive measure ANOVA was carried out on the experimental results. The independent variables were Time, Group, and SRT condition (NV, VD), while the dependent variables were COPx, COPy, TTW, and EA results. A 2 × 2 × 2 three-way repetitive measure ANOVA was constructed.

The findings were as follows:

1. **COP*_*x*_*:**
  • Group effect: Significant, showing reduced displacement in the experimental group post-intervention (*F*_(1,16)_ = 5.641, *P* < 0.05, η*_*p*_*^2^ = 0.261).
  • Time effect: Significant improvement post-experiment (*F*_(1,16)_ = 33.741, *P* < 0.01, η*_*p*_*^2^ = 0.678).
  • SRT condition effect: COP*_*x*_* was higher under VD than NV conditions (*F*_(1,16)_ = 17.841, *P* < 0.001, η*_*p*_*^2^ = 0.527).
2. **COP*_*y*_*:**
  • No significant Group effect (*F*_(1,16)_ = 0.071, *P* = 0.794, η*_*p*_*^2^ = 0.004).
  • Significant improvement over Time (*F*_(1,16)_ = 61.522, *P* < 0.001, η*_*p*_*^2^ = 0.794).
  • Higher values observed under VD than NV (*F*_(1,16)_ = 20.233, *P* < 0.001, η*_*p*_*^2^ = 0.558).
3. **TTW:**
  • No significant Group effect (*F*_(1,16)_ = 0.004, *P* = 0.949, η*_*p*_*^2^ = 0).
  • Marked improvement over Time (*F*_(1,16)_ = 292.575, *P* < 0.001, η*_*p*_*^2^ = 0.948).
  • Higher values under VD conditions (*F*_(1,16)_ = 67.519, *P* < 0.001, η*_*p*_*^2^ = 0.808).
4. **EA:**
  • No significant Group effect (*F*_(1,16)_ = 0.097, *P* = 0.76, η*_*p*_*^2^ = 0.006).
  • Significant improvement over Time (*F*_(1,16)_ = 36.433, *P* < 0.001, η*_*p*_*^2^ = 0.998).
  • Elevated values under VD conditions (*F*_(1,16)_ = 240.745, *P* < 0.001, η*_*p*_*^2^ = 0.938).

The repetitive measure ANOVA results highlighted that the main effects of Time and SRT condition were significant across all four dependent variables. The experimental results affirmed that the experimental design in this study achieved the expected value. Specifically, when VD was treated as an independent variable, the results revealed an improvement in the balance ability of participants in both groups under the NV task. However, the performance of the experimental group, under the SIT intervention during the VD task, was more resilient to interference, thereby showcasing a more stable performance.

**FIGURE 5:**
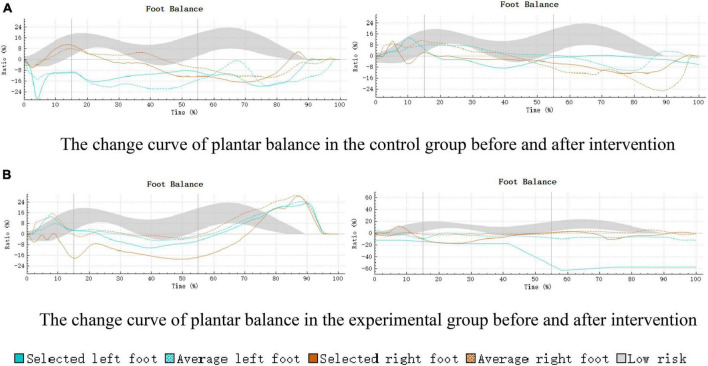
Curve of plantar balance changes in control and experimental groups pre- and post-intervention. **(A)** The change curve of plantar balance in the control group before and after intervention. **(B)** The change curve of plantar balance in the experimental group before and after intervention.

The acquired plantar balance results were visualized through the 3D scan view of Footscan 9^®^ (Figure 5). The balance change curve was in alignment with the results of this study, further attesting to the effectiveness of SIT intervention in enhancing the balance function of children with ASD.

### 3.2. Behavioral measures from the Go/No-Go fNIRS session

The behavioral indices for the Go/No-Go tasks encompassed reaction time (RT) and accuracy ratio (AR). Preliminary data processing results are depicted in the descriptive statistics found in Table 3.

**TABLE 3:** Behavioral data from Go/No-Go tasks in participants, RT stands for reaction time; AR stands for accuracy ratio.

		Control group		Experimental group	
Index		Go	No-Go	Go	No-Go
RT (ms)	Pre	425.74 ± 58.28	432.04 ± 47.02	422.61 ± 43.12	438.47 ± 47.15
	Post	387.23 ± 49.31	391.30 ± 48.91	375.42 ± 53.17	382.19 ± 49.68
AR (%)	Pre	0.89 ± 0.03	0.85 ± 0.02	0.87 ± 0.16	0.84 ± 0.09
	Post	0.91 ± 0.08	0.88 ± 0.15	0.92 ± 0.11	0.89 ± 0.10

The main and interaction effects among all indices underwent analysis through repeated measure ANOVA using SPSS. Results revealed significant main effects for both RT, *F*_(1,16)_ = 193.85, *P* < 0.001, η2 = 0.93, and AR, *F*_(1,16)_ = 449.42, *P* < 0.001, η*_*p*_*^2^ = 0.95, when considered as independent variables. This finding implies an interaction between RT and AR in Go/No-Go tasks, further validating the suitability of the selected Go/No-Go cognitive neural task for this experiment.

**FIGURE 6:**
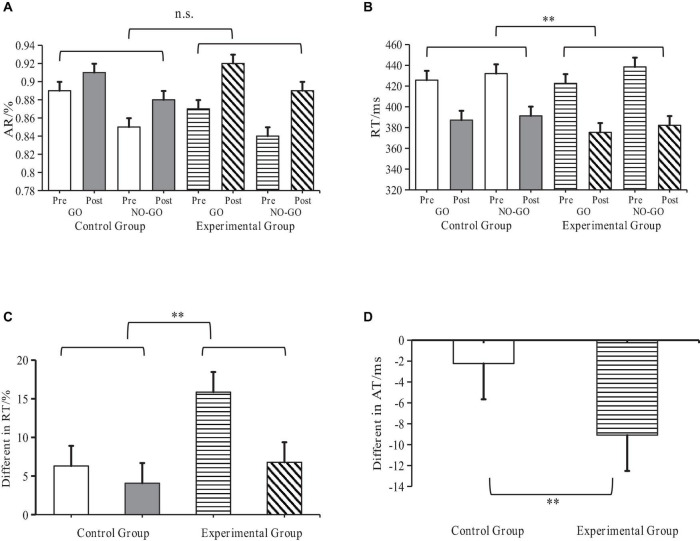
Behavioral performance of ASD children and Go/No-Go task effects. **Indicates *p* < 0.01; n.s. indicates *p* > 0.05; AR denotes accuracy ratio; RT denotes reaction time; ASD denotes autism spectrum disorder. **(A)** RT Changes at each task level. **(B)** AR Changes at each task level. **(C)** Difference in Go/No-Go effects of RT before and after **(D)** Inter-task difference in Go/No-Go effects of RT.

Pre- and post-experiment data from both groups underwent analysis through a *t*-test to compare the RT differences between experimental and control groups. The findings demonstrated that the RT of the experimental group was significantly lower than that of the control group (*P* < 0.01), further suggesting that SIT intervention can enhance the performance level of cognitive neural tasks in children with ASD (Figure 6).

### 3.3. Functional near-infrared spectroscopy (fNIRS) results

Data pre-processing occurred before data analysis: linear polynomial fitting and band-pass filtering were performed on the oxy-Hb signals from each channel. Signal wavebands demonstrating substantial fluctuations in the baseline region and during the Go/No-Go tasks were removed, and a waveband frequency of 0.01–0.8 Hz was selected to minimize data bias caused by cardiac pulse, natural breathing, and head movement noise.

Post-processing, the data from Go/No-Go tasks were categorized into GO and NO-GO task data. Although fNIRS can record data on three blood oxygen signals – oxy-Hb, deoxy-Hb, and total Hb, the analysis focused on oxy-Hb signal changes due to their higher sensitivity to cerebral blood flow changes, neural activation levels in the prefrontal lobe, signal-to-noise ratio, and test-retest reliability (Sato et al., 2004; Uludag et al., 2004; Zhang et al., 2011).

**FIGURE 7:**
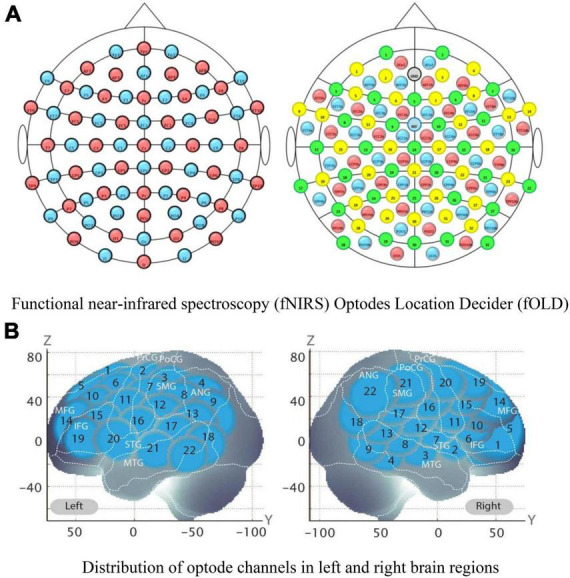
Functional near-infrared spectroscopy (fNIRS) optode channels and activated regions of interest (ROIs) division in brain regions. The location distribution map of functional near-infrared spectroscopy (fNIRS) optodes, and the prefrontal lobe and several channels formed an activated region of interest (ROI); **(A)** Functional near-infrared spectroscopy (fNIRS) Optodes Location Decider (fOLD). **(B)** Distribution of optode channels in left and right brain regions. The schematic diagram of 44 channels on the Montreal Neurological Institute (MNI) space coordinate axes.

During the Go/No-Go tasks, high activation was noted in the right inferior frontal gyrus (R-IFG) and rostral middle frontal gyrus (R-MFG). Therefore, these regions were selected as the study’s regions of interest (ROI) (Figure 7).

A repeated measure ANOVA tested the main effect of all independent variables and interactions between all dependent variables. Tests were performed on oxy-Hb concentration, and the SIT and Go/No-Go effects in the two ROIs. The results revealed significant interactions between SIT and Go/No-Go effects when considering the oxy-Hb concentration in the R-IFG, *F*_(1,16)_ = 18.02, *P* < 0.001, η^2^ = 0.55, and R-MFG regions, *F*_(1,16)_ = 16.50, *P* < 0.001, η^2^ = 0.52, as independent variables. This outcome further affirms that SIT can effectively elevate oxy-Hb blood oxygen concentration in the aforementioned brain regions.

**FIGURE 8:**
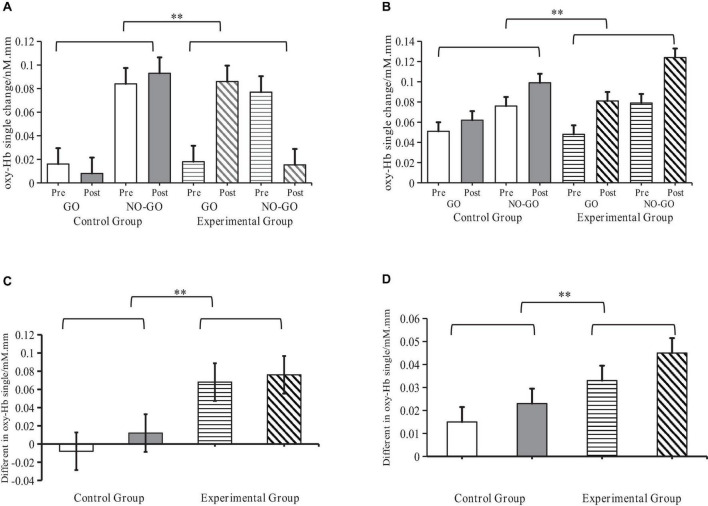
Levels of activation of oxy-Hb signals by sensory integration training (SIT) in ASD children. **Indicates *P* < 0.01; oxy-Hb denotes oxyhemoglobin; R-IFG denotes right inferior frontal gyrus; R-MFG denotes rostral middle frontal gyrus. **(A)** Changes of oxy-Hb signals in the R-IFG brain region **(B)** Changes of oxy-Hb signals in the R-MFG brain region. **(C)** Difference in Go/No-Go effects in the R-IFG brain region. **(D)** Difference in Go/No-Go effects in the R-MFG brain region.

A paired sample *t*-test further compared the differences in oxy-Hb signals between experimental and control groups before and after the Go/No-Go effects test. Results showed that in both the R-IFG, *t*_(1,16)_ = 4.25, *P* < 0.001, *r* = 0.55, and R-MFG regions, *t*_(1,16)_ = 4.06, *P* < 0.001, *r* = 0.52, oxy-Hb signals in the experimental group were significantly higher than in the control group. This suggests that SIT significantly enhances neural activation in the R-IFG and R-MFG regions (Figure 8).

## 4. Discussion

### 4.1. The meaning of SRT and Footscan results

Firstly, our findings indicate that, compared to traditional therapies, Sensory Integration Therapy (SIT) significantly enhances the balance capabilities of children with autism spectrum disorder (ASD). More specifically, biomechanical evidence derived from Footscan reveals that while traditional therapy showed some improvement in participants’ balance under the SRT’s NV task conditions, it failed to demonstrate efficacy in VD task scenarios. For SIT, however, improvements in participants’ balance were observed under both NV and VD conditions, with the enhancements being particularly pronounced under VD tasks. Under typical visual circumstances, children with ASD predominantly rely on visual signals transmitted to the cerebral cortex. They integrate visual and proprioceptive systems with various bodily components to execute a range of intricate movements. Throughout this process, vision stands out as the most vital information conduit (Little, 2018; Lim et al., 2020). When deficits in sensory integration led to visual impairments, the stability and balance of posture become correspondingly impacted (Cornilleau-Pérès et al., 2005; Stins et al., 2009). Molloy et al. (2003) discovered that children with ASD encounter more significant challenges in balance tasks deprived of visual cues compared to typically developing (TD) children (Molloy et al., 2003). This underscores an excessive dependence on visual cues among children with ASD. Two subsequent studies further corroborated the visual reliance observed in children with ASD. These studies indicated that, when subjected to visual deprivation, children with ASD demonstrated greater postural sway during a visual searching task (Stins et al., 2015) compared to an auditory digit span task (Memari et al., 2014). Such postural swaying is attributed to the inadequate integration of vestibular, proprioceptive, and visual systems (Molloy et al., 2003; Minshew et al., 2004). Utilizing SIT for intervention, we integrated the seven sensory systems of children with ASD, facilitating coordinated completion of numerous physical activities. The results highlight that the enhanced balance capabilities weren’t solely a result of visual dependence. Intriguingly, SIT seemed to induce a compensatory effect on the sensory input deficiencies in children with ASD, particularly in vision, vestibular perception, and proprioception. This emergent compensatory effect warrants further in-depth exploration in future research.

On another note, the heterogeneity and frequent comorbidities associated with ASD contribute to the intricacy of its etiology. Leo Kanner, renowned as one of the most influential psychiatrists of the 20^th^ century and often dubbed the father of child psychiatry, established as early as 1943 through clinical observations that children with ASD typically show motor skill impairments (Kanner, 1943). In 2014, Professor Lai Mengquan of the University of Toronto’s Psychiatry Department published an article titled “Autism” (Lai et al., 2014) in The Lancet. In this piece, he reaffirmed the prevalent motor skill challenges among individuals with ASD. Following research has empirically indicated (Gowen and Hamilton, 2013) that children with ASD face delays in both fine and gross motor development, often demonstrating uncharacteristic and aberrant motor patterns. Whyatt and Craig (2012) undertook a study evaluating the factors influencing the motor capabilities of children with ASD (Whyatt and Craig, 2012). Their results suggest that balance abilities are the most prominently affected skills among the various motor challenges faced by children with ASD. The effectiveness of balance ability heavily relies on the quality of posture control. Relevant studies indicate that compared to typically developing children, children with ASD exhibit specific deficits in static postural control (Wang et al., 2016). This, to some extent, highlights the challenges faced by children with ASD in various control mechanism movements, potentially impacting their daily life and physical activities. The issues stem from abnormalities in the cortical cerebellar brain structure network impacting the balance function of postural control. The cerebellum holds a key role in orchestrating movement, encompassing guided feedback and reactive behavioral correction during movement (Baev et al., 2002), and synchronizing the timing and spatial coordination of different body joints (Whitney et al., 2008). Our findings suggest that SIT can enhance the balance abilities of children with ASD by mediating corresponding postural control mechanisms via various brain regions (Palliyath et al., 1998). However, SIT is not only associated with the balance abilities of children with ASD but also holds a significant correlation with visuomotor integration, cognitive abilities, motor skills, and postural control. The intrinsic mechanisms of its action require extensive and repeated experimental validation in subsequent studies.

### 4.2. The meaning of Go/No-Go tasks and fNIRS results

Furthermore, evidence from the Go/No-Go task highlights that children with ASD undergoing SIT demonstrate a notable reduction in RT, pointing to enhanced response efficiency and faster cognitive processing. Conversely, the rise in AR emphasizes the favorable effects of SIT on response accuracy and precision, signifying improvements in participants’ executive functions. In tandem with this, fNIRS neurology findings corroborate the underlying neural pathways through which SIT optimizes the executive functions in children with ASD.

These findings suggest that SIT may have beneficial effects on executive function in ASD children by modulating neural activation in the PFC. One possible mechanism is that SIT may improve sensory processing and integration in ASD children, which may in turn facilitate cognitive processing and regulation. Sensory processing refers to the ability to receive, organize, and interpret sensory information from the environment and the body (Ayres, 1972). Sensory integration refers to the ability to use sensory information to plan and execute adaptive responses (Ayres et al., 2005). ASD children often exhibit sensory processing and integration difficulties, such as hypersensitivity or hyposensitivity to sensory stimuli, sensory seeking or avoiding behaviors, and poor sensory discrimination or modulation (Tomchek and Dunn, 2007). These difficulties may impair their attention, perception, memory, learning, and emotion regulation (Baranek, 2002).

Addressed concretely, we focused on two subregions of the PFC: the right inferior frontal gyrus (R-IFG) and the rostral middle frontal gyrus (R-MFG). The R-IFG is implicated in inhibitory control, as it inhibits prepotent responses and resolves response conflicts (Aron et al., 2014). The R-MFG is involved in working memory, cognitive flexibility, and attentional control, as it maintains task-relevant information and switches between different task rules or strategies (Derrfuss et al., 2005). Our results showed that SIT significantly increased oxy-Hb signals in both R-IFG and R-MFG regions during Go/No-Go tasks, indicating enhanced neural activation and blood flow in these regions. Moreover, our behavioral results showed that SIT significantly reduced reaction time and increased accuracy ratio during Go/No-Go tasks, indicating improved performance and efficiency in executive function. By enhancing neural activation in the R-IFG and R-MFG regions during Go/No-Go tasks, SIT may improve inhibitory control and cognitive flexibility among ASD children through these pathways. Inhibitory control is crucial for suppressing prepotent responses or distractors during Go/No-Go tasks, such as: they were instructed to quickly press the space bar when they saw an elephant, whereas for other animals, they needed to suppress their impulses. Cognitive flexibility is essential for switching between different task rules or stimuli during Go/No-Go tasks, such as: the different animals in this study. By improving performance and efficiency in Go/No-Go tasks, SIT may improve behavioral and functional outcomes among ASD children.

SIT is based on the theory that providing appropriate and individualized sensory stimulation can enhance neural plasticity and promote optimal sensory processing and integration (Schaaf and Miller, 2005). SIT involves various activities that challenge the vestibular, proprioceptive, tactile, auditory, and visual systems, such as swinging, bouncing, spinning, jumping, crawling, balancing, touching, listening, and looking (Bundy and Lane, 2019). SIT aims to improve sensory modulation, discrimination, praxis, and organization skills in ASD children, which may lead to better behavioral and functional outcomes (Parham et al., 2011).

By improving sensory processing and integration in ASD children, SIT may also improve executive function through several pathways. First, SIT may enhance motor coordination and balance function in ASD children, which may in turn improve their inhibitory control and cognitive flexibility. Previous studies have shown that motor skills and executive function are closely related in both typical and atypical development (Diamond, 2000; Hill, 2001; Piek et al., 2008). Motor skills require inhibitory control to suppress unwanted movements and cognitive flexibility to adapt to changing environments or task demands (Best and Miller, 2010). Conversely, executive function requires motor skills to execute planned actions and monitor feedback (Diamond, 2013). Therefore, SIT may improve executive function by improving motor skills in ASD children.

On the other hand, SIT may enhance attentional control and working memory in ASD children, which may in turn improve their inhibitory control and cognitive flexibility. Attentional control refers to the ability to selectively focus on relevant stimuli or tasks while ignoring irrelevant distractors (Posner and Petersen, 1990). Working memory refers to the ability to temporarily store and manipulate information for complex cognitive tasks (Baddeley, 2003). Both attentional control and working memory are essential for executive function, as they enable goal-directed behavior and problem-solving (Miyake et al., 2000). SIT may improve attentional control and working memory in ASD children by providing optimal levels of arousal and stimulation that match their individual sensory preferences and needs (Schaaf et al., 2014). SIT may also improve attentional control and working memory by enhancing interoception, which is the sense of the internal state of the body (Craig, 2002). Interoception is important for attentional control and working memory, as it helps regulate emotional states, arousal levels, motivation, and self-awareness (Critchley and Garfinkel, 2017).

This study has several implications for the theory and practice of SIT for ASD children. First, this study provides empirical evidence for the effectiveness and potential benefits of SIT on balance function and executive function in ASD children, using objective and quantitative measures. Previous studies on SIT have been mostly qualitative or based on subjective measures, which have limited validity and reliability. This study uses Footscan and fNIRS technologies to measure biomechanical and neurophysiological outcomes of SIT intervention, which enhances the credibility and rigor of the research. Second, this study demonstrates a novel and multidisciplinary approach to assess the effects of SIT on ASD children, by integrating biomechanics, hemodynamics, and cognitive neuroscience. This approach allows us to explore the underlying mechanisms of SIT intervention from multiple perspectives and levels of analysis, which may reveal new insights and discoveries that could not be obtained from a single discipline or method. Third, this study contributes to the existing literature on SIT for ASD by providing a comprehensive and critical discussion of the findings, highlighting the implications, limitations, and future directions of the research. This discussion may help researchers and practitioners to better understand the strengths and weaknesses of SIT intervention, as well as the gaps and challenges that need to be addressed in future studies.

### 4.3. Implications, limitations, and future directions

This study also has several limitations that need to be acknowledged and addressed in future research. First, the sample size is relatively small (*n* = 18) and lacks diversity (all Chinese, mostly male, right-handed). A larger and more representative sample would increase the statistical power and external validity of the study. Future studies should recruit more participants from different backgrounds, genders, handedness, and severity levels of ASD. Second, the control group received a different intervention (treatment as usual) than the experimental group (SIT), which may introduce confounding factors and bias. A better control condition would be a sham or placebo intervention that mimics the SIT without its active ingredients. Future studies should use a randomized controlled trial design with a proper control group to eliminate potential confounds and establish causal inference. Third, the study did not provide sufficient details on how the SIT intervention was implemented, such as the frequency, duration, intensity, content, and individualization of the sessions. A clear description of the intervention protocol would enhance the replicability and transparency of the study. Future studies should provide more information on how SIT intervention was delivered and tailored to each participant’s needs and preferences. Fourth, the study did not report any measures of adherence, satisfaction, or adverse effects of the intervention, which are important indicators of feasibility and acceptability of SIT for ASD children and their families. Future studies should collect and analyze data on these aspects to evaluate the practicality and safety of SIT intervention.

In addition to addressing these limitations, future studies should also explore other aspects of SIT intervention that were not covered in this study. For example, future studies should measure other aspects of cognitive function and quality of life in ASD children that may be affected by SIT intervention, such as attention span, memory capacity, language skills, social skills, and self-esteem.

## 5. Conclusion

This study advances our understanding of Sensory Integration Therapy (SIT) as a valuable intervention for children with autism spectrum disorder (ASD), improving both balance and executive function. SIT, in comparison to routine interventions, shows more pronounced and targeted impacts.

## Data availability statement

The original contributions presented in this study are included in the article/supplementary material, further inquiries can be directed to the corresponding author.

## Ethics statement

The studies involving humans was conducted in accordance with the Declaration of Helsinki, and approved by the Human Experimental Ethics Inspection of Guangzhou Sport University (ethics number: 2022LCLL-21, date of approval: 7 July 2022). The studies were conducted in accordance with the local legislation and institutional requirements. Written informed consent for participation in this study was provided by the participants’ legal guardians/next of kin. Written informed consent was obtained from the individual(s) for the publication of any potentially identifiable images or data included in this article.

## Author contributions

JD: Data curation, Formal analysis, Methodology, Visualization, Writing – original draft, Writing – review and editing. TL: Data curation, Formal analysis, Investigation, Methodology, Writing – original draft, Writing – review and editing. XD: Conceptualization, Funding acquisition, Methodology, Supervision, Writing – review and editing.
